# Cardiovascular risk in renal transplant recipients

**DOI:** 10.1007/s40620-018-0549-4

**Published:** 2018-11-07

**Authors:** Paul A. Devine, Aisling E. Courtney, Alexander P. Maxwell

**Affiliations:** 1 Regional Nephrology and Transplant Unit, Belfast City Hospital Northern Ireland, Belfast, BT9 7AB UK; 2 Centre for Public Health, Queen’s University Belfast, Belfast, UK

**Keywords:** Kidney transplant, Cardiovascular disease, Risk factors, Immunosuppression, Biomarkers

## Abstract

Successful kidney transplantation offers patients with end-stage renal disease the greatest likelihood of survival. However, cardiovascular disease poses a major threat to both graft and patient survival in this cohort. Transplant recipients are unique in their accumulation of a wide range of traditional and non-traditional cardiovascular risk factors. Hypertension, diabetes, dyslipidaemia and obesity are highly prevalent in patients with end-stage renal disease. These risk factors persist following transplantation and are often exacerbated by the drugs used for immunosuppression in organ transplantation. Additional transplant-specific factors such as poor graft function and proteinuria are also associated with increased cardiovascular risk. However, these transplant-related factors remain unaccounted for in current cardiovascular risk prediction models, making it challenging to identify transplant recipients with highest risk. With few interventional trials in this area specific to transplant recipients, strategies to reduce cardiovascular risk are largely extrapolated from other populations. Aggressive management of traditional cardiovascular risk factors remains the cornerstone of prevention, though there is also a potential role for selecting immunosuppression regimens to minimise additional cardiovascular injury.

**Electronic supplementary material:**

The online version of this article (10.1007/s40620-018-0549-4) contains supplementary material, which is available to authorized users.

## Introduction

End-stage renal disease (ESRD) is an irreversible condition which results in a significant reduction in individual life expectancy and has major treatment costs. Options for renal replacement therapy (RRT) are limited to different modalities of dialysis or kidney transplantation. Transplantation is considered the optimal form of RRT due to the numerous benefits conferred by a functioning allograft compared to dialysis. Chief among these are increased patient survival and improved quality of life [1, 2]. However, transplantation is not a panacea for the many metabolic derangements brought about by ESRD. Despite significant surgical and immunopharmacological advancements, renal transplant recipients (RTR) do not share the life expectancy of their age-matched peers. While a healthy 20-year-old individual in Europe can expect to live a further 62 years, the same individual can expect to live 44 years following a successful renal transplant and only 22 years upon commencing chronic dialysis [3].

This discrepancy in life expectancy is at least partially attributable to the increased incidence of cardiovascular disease that occurs in patients with ESRD. The risk of cardiovascular disease in patients undergoing regular haemodialysis is estimated to be 10–20 times higher than that of the general population [4]. While a successful transplant can reduce this risk significantly, RTR still have an annual cardiovascular event rate of 3.5–5% [5]. Accordingly, cardiovascular disease remains one of the leading causes of death in RTR [6, 7]. As death with a functioning graft is the leading cause of graft loss [8], potential strategies to successfully reduce the burden of cardiovascular disease are essential for improving both graft and patient outcomes.

In this review, we provide insights into both traditional and non-traditional factors which interact to contribute to the increased risk of cardiovascular disease in RTR. The challenges posed by identifying those RTR at highest cardiovascular risk are emphasised and some of the potential measures that may be employed to reduce this risk are described.

## Epidemiology

In comparison to their age-matched peers, RTR display a three- to five-fold increased risk of cardiovascular disease [9]. Over 50% of cardiovascular-related deaths in RTR are sudden, and presumed to be secondary to cardiac arrhythmia and cardiac arrest [7]. Thus, non-atherosclerotic cardiac disease appears to be of particular importance in this cohort. This likely represents the high burden of structural cardiac abnormalities, such as myocardial fibrosis and left ventricular hypertrophy (LVH), present in patients with ESRD commencing RRT [10].

The clinical pattern of cardiovascular disease observed in RTR is otherwise broadly similar to that observed in non-transplanted individuals. The incidence of myocardial infarction (MI) is high with rates of 5.6% and 11.1% at 1 year and 3 years post-transplantation, respectively [11]. This is approximately six-fold higher than observed in the general population [11]. Congestive heart failure is also an important cause of cardiovascular mortality [7, 12].

Registry data from the USA have consistently identified cardiovascular disease as the leading cause of death in RTR [7]. However, recent data from the UK Renal Registry demonstrate that the annual mortality directly attributable to cardiovascular disease in RTR has fallen over the last decade [6]. Despite this, cardiovascular disease remains a significant clinical problem and is responsible for approximately 20–35% of mortality in RTR [6, 7].

## Traditional cardiovascular risk factors

The first results of the Framingham Heart Study published in 1957 identified hypercholesterolaemia and hypertension as risk factors for the development of cardiovascular disease [13]. Subsequently smoking, obesity and diabetes mellitus were identified as being important. These “traditional” cardiovascular risk factors are also strongly associated with the development of chronic kidney disease (CKD). Their prevalence is therefore markedly increased among RTR, who may have had a prolonged duration of accelerated cardiovascular risk prior to transplantation (Table 1) [14, 15].

**Table 1:** Cardiovascular risk factors in renal transplant recipients</tb>

Traditional risk factors	Non-traditional risk factors
Hypertension^a,b^	Renal impairment (reduced eGFR)
Diabetes mellitus^a,b^	Proteinuria
Cigarette smoking	Left ventricular hypertrophy
Dyslipidaemia^a,b^	Anaemia
Obesity^a^	Acute rejection episodes

^a^Exacerbated by steroid use

^b^Exacerbated by calcineurin inhibitor use

### Hypertension

Hypertension is already present in over 60% of patients prior to transplantation [14]. It usually persists post-transplant and is exacerbated by some immunosuppressive drugs e.g. calcineurin inhibitors [16]. Other potential contributory factors include graft dysfunction and transplant renal artery stenosis. The prevalence of hypertension in RTR is reportedly greater than 70% [17].

Hypertension is associated with graft failure, cardiovascular disease and mortality [18, 19]. Kasiske and colleagues found that each 10 mmHg increase in systolic blood pressure (SBP) is associated with an 18% increase in the risk of death in RTR [18].

A recent study by Mallamaci and co-workers highlighted the prognostic significance of ambulatory blood pressure monitoring (ABPM) in RTR [20]. They demonstrated that the prevalence of nocturnal hypertension is greater than double that of daytime hypertension in RTR, and that nocturnal blood pressure is a stronger predictor of graft failure than daytime values [20]. ABPM is particularly useful in identifying masked hypertension, which is a common problem in this population [21].

The optimal blood pressure target in RTR remains unclear due to a lack of prospective trials. Recommendations are often based on observational and retrospective data. The Collaborative Transplant Study showed that risk of cardiovascular mortality and graft failure is reduced when SBP is < 140 mmHg at 3 years post-transplant [22]. Another study by the same group demonstrated that SBP < 120 mmHg is associated with improved graft survival compared to SBP < 130 mmHg [23]. Current KDIGO guidelines recommend a target blood pressure of < 130/80 mmHg for all RTR, whereas guidelines in the UK set a target of < 140/90 mmHg in the absence of proteinuria [24, 25].

The existing evidence-base is insufficient to recommend the use of one antihypertensive agent over another in RTR. Clearly there are circumstances in which one particular class of drug may be more desirable. Calcium channel blockers can counteract the vasoconstrictive effects of calcineurin inhibitors [16]. ACE-inhibitors (ACEi) and angiotensin-receptor blockers (ARB) are useful in reducing proteinuria and can ameliorate post-transplant erythrocytosis, (which has been postulated to potentially be of relevance), though their use in the very early post-transplant period requires close monitoring [24–26]. Irrespective of the choice of antihypertensive drug(s), the aim should be to achieve adequate blood pressure control to improve graft and patient survival.

### Diabetes mellitus

Diabetes mellitus is highly prevalent in RTR. In Europe, 26% of patients with ESRD have diabetic nephropathy registered as their primary renal disease [27]. A proportion of these patients proceed to transplantation. In addition, RTR without pre-existing diabetes are at risk of developing post-transplantation diabetes mellitus (PTDM).

Kasiske and colleagues demonstrated that for RTR with pre-existing diabetes, the risk of cardiovascular disease and stroke is increased threefold compared to non-diabetic recipients [28]. The adjusted risk of peripheral vascular disease is also up to 28 times higher [28]. This heightened risk may reflect progression of subclinical cardiovascular disease which was present at the time of transplantation but not identified during work-up. In a study by Ramanathan and co-workers of patients with ESRD, 33% of asymptomatic individuals with type 1 diabetes and 48% of persons with type 2 diabetes had significant coronary artery stenosis at angiogram [29]. Despite this, there is currently insufficient evidence to support routine use of coronary angiogram in the work-up for transplantation of asymptomatic individuals with diabetes.

PTDM is also strongly associated with increased cardiovascular risk in RTR [30]. Established risk factors include deceased donor graft, older recipient age, recipient ethnicity (Hispanic), recipient race (black) and the presence of hypertension, obesity or substantial post-transplant weight gain. Use of calcineurin inhibitors and steroids also contribute to PTDM risk by blunting insulin secretion and increasing insulin resistance [31]. Screening for PTDM is recommended at each clinic visit using dipstick urinalysis and blood glucose measurement. Formal diagnosis should be based on WHO criteria using HbA1c, fasting blood glucose level or the oral glucose tolerance test when the recipient is clinically stable [25].

The management of diabetes in RTR can be challenging and often requires specialist input from a diabetologist [25]. In early post-transplant hyperglycaemia, insulin is often required to counteract the effects of high-dose steroids [32]. However, in the outpatient setting, lifestyle measures such as weight loss and use of oral hypoglycaemic agents are appropriate initial steps. There is currently a dearth of evidence on the efficacy or safety of newer oral agents, such as sodium and glucose co-transporter 2 (SGLT2) inhibitors and glucagon-like peptide 1 (GLP-1) agonists in RTR. As a result, an international consensus meeting in 2013 was unable to support the introduction of a hierarchy of hypoglycaemic agents for PTDM management [32]. Their recommendation was to alter the immunosuppressive regimen and introduce hypoglycaemic agents in an approach individualised to the patient.

However, studies in this field are ongoing (see Table 2). If demonstrated to be safe, the potential cardiovascular and renal protection offered by SGLT2 inhibitors and GLP-1 agonists make them an appealing choice for RTR [33].

**Table 2:** Active/planned studies related to post-transplant diabetes mellitus in renal transplant recipients, as registered on *clinicaltrials.gov*

Identifier	Study title	Design	Description	Expected completion
NCT03642184	Efficacy and Safety of Empagliflozin in NODAT	Open-label RCT	Investigation of the safety and clinical effects of empagliflozin compared with linagliptin in RTR. Outcomes include renal parameters and glycaemic control	December 2020
NCT02083991	Trial of Steroid Avoidance and Low-dose CNI by ATG-induction in Renal Transplantation (SAILOR)	Open-label RCT	Evaluation of the effects of a steroid-free, low-dose CNI immunosuppression regimen on the incidence of PTDM in RTR	January 2017
NCT03113110	Empagliflozin in Post-Transplantation Diabetes Mellitus (EMPTRA-DM)	Phase 2 non-inferiority study	Conversion of RTR with PTDM from insulin (< 40 units per day) to empaglifozin. Outcomes include glycaemic control, weight and blood pressure	December 2019
NCT01928199	Efficacy Study of Sitagliptin to Prevent New-onset Diabetes After Kidney Transplant	Phase 2, double-blind RCT	Evaluation of the efficacy of sitagliptin compared with placebo at reducing the incidence of PTDM in RTR who develop hyperglycaemia within 72 h of transplantation	December 2019
NCT02558452	European Transplant Registry of Senior Renal Transplant Recipients on Advagraf (SENIOR)	Observational (patient registry)	Establishment of a registry of RTR > 65 years old initially treated with once-daily tacrolimus, mycophenolate and steroids. Long-term (10 year) outcomes include graft loss, death, cardiovascular events and development of PTDM	January 2028
NCT02849899	Prevention of Diabetes After Transplantation by Vildagliptin in the Early Post-transplant Period (PRODIG)	Triple-blind RCT	Comparison of the efficacy vildagliptin versus placebo in reducing the incidence of PTM 1 year post-transplantation	January 2020

As of September 2018

Search terms; “post-transplant diabetes mellitus”, “NODAT”. Studies focused on non-renal transplantation were excluded

*NODAT* new-onset diabetes after transplantation, *RCT* randomized controlled trial, *RTR* renal transplant recipients, *CNI* calcineurin-inhibitor, *PTDM* post-transplant diabetes mellitus

### Dyslipidaemia

Dyslipidaemia is a common problem following transplantation, with over 60% of RTR affected [34]. Transplantation is associated with elevations of total cholesterol, LDL-cholesterol and triglycerides, largely due to immunosuppression regimens. Steroids, calcineurin inhibitors and mTOR inhibitors all have deleterious effects on lipid concentrations [35].

There is a strong association between dyslipidaemia and cardiovascular disease in RTR. The risk of ischaemic heart disease is doubled with serum cholesterol > 200 mg/dL or triglycerides > 350 mg/dL [15]. Additionally, total cholesterol concentration at 1-year post-transplant independently predicts mortality in RTR [28].

Screening for dyslipidaemia should be undertaken early following transplantation and then at least annually [24, 25]. Management can include reduction of immunosuppression doses or, where relevant, switching from ciclosporin to tacrolimus [36]. However, guidelines now recommend the use of HMG-CoA reductase inhibitors (statins) in RTR with hypercholesterolaemia [24, 25]. These are based on evidence from the ALERT trial [12], which showed fluvastatin successfully lowered LDL-cholesterol by 32%. Although the trial was inadequately powered for its primary composite endpoint (major adverse cardiac events, MACE), fluvastatin reduced the risk of cardiac death and non-fatal MI by 35% [12]. Results from a post hoc analysis demonstrated that risk reduction was greatest when statin therapy was introduced within the first 2 years following transplantation [37]. Despite this evidence, uncertainty over target levels, poor tolerance of statin therapy in a substantial minority of patients, and the concern regarding polypharmacy, impacts on the widespread clinical prescription of statin therapy in this cohort.

Proprotein convertase subtilisin kexin type 9 (PCSK9) inhibitors have recently been used as adjunctive therapy to statins in patients who fail to achieve adequate cholesterol control [38]. These monoclonal antibodies lower cholesterol through their action on LDL-receptors in the liver, increasing LDL uptake from the blood. In the FOURIER trial, evolocumab significantly lowered LDL-cholesterol concentration and reduced the incidence of major cardiovascular events in patients already taking statins [39]. As a novel drug class, there is no experience of PCSK9 inhibitor use in RTR. However, it is important to note that the FOURIER trial specifically excluded transplant recipients and those with advanced renal impairment. Therefore, further trials which include such patients are required before use of PCSK9 inhibitors can be considered in RTR.

### Cigarette smoking

Kasiske and Klinger published a study in 2000 reporting that 25% of recipients smoked at the time of transplantation. The smoking prevalence in RTR mirrored the prevalence in the general population [40]. This study demonstrated that smoking was an independent risk factor for graft loss, cardiovascular disease and death [40]. A recent post hoc analysis of the FAVORIT study demonstrated similar results, with continued smoking increasing the risk of all-cause mortality by 70% [41]. There are insufficient interventional trials investigating the efficacy of smoking cessation methods in RTR. However, methods used in the general population are likely to be safe and should be used [25].

Interestingly, the study by Kasiske and Klinger demonstrated that smoking cessation more than 5 years prior to transplantation significantly reduced the risk of adverse outcomes [40]. This suggests that smoking cessation strategies would be most effectively used in patients with CKD before they ever reach ESRD.

### Weight gain and obesity

The global obesity epidemic is reflected in the renal transplant population. Recent data showed that approximately 35% of RTR in the USA are obese at the time of transplantation with a body mass index (BMI) ≥ 30 kg/m^2^. This figure is increasing annually as practice evolves to include higher risk recipients on the waiting list. No definitive safe upper limit for BMI at time of transplantation has been established [42]. Obese ESRD patients who are transplanted have improved survival rates compared to those who remain on dialysis [43]. In the current era, their risk of graft failure and death is comparable to those who undergo transplantation with a normal BMI [44].

Nevertheless, obesity is associated with cardiovascular disease. A study by Lentine and co-workers demonstrated that the risk of cardiac disease, particularly heart failure and atrial fibrillation, is increased by 25% for each 5 unit increase in recipient BMI [45]. Obesity is associated with hypertension, dyslipidaemia, impaired glucose tolerance and proteinuria in RTR, all of which increase cardiovascular risk [46]. A study by Johnson and colleagues revealed that weight gain is a common issue post-transplantation, with over half of RTR gaining more than 10% body weight [47]. Post-transplantation weight gain is also associated with adverse outcomes, even in the absence of obesity [47].

Weight loss may be aided with exercise and appropriate dietary advice, though there are no clinical trials to support this in RTR specifically. The use of pharmacological interventions to stimulate weight loss is not currently recommended due to concerns these may interfere with the absorption of immunosuppressive drugs [25]. Steroid reduction or withdrawal may appear intuitive but the effect on obesity is limited [48].

## Non-traditional cardiovascular risk factors

With regards to cardiovascular risk, individuals with CKD and ESRD have unique characteristics given the combined effects of prolonged exposure to traditional risk factors compounded by the accumulated burden of non-traditional risk factors related to CKD and ESRD (Table 3). A functioning allograft can mitigate the impact of some of these non-traditional factors, but they typically persist to some degree following transplantation.

**Table 3:** Summary of studies relevant to the risk of cardiovascular disease in renal transplant recipients

Area of study	Types of studies	Reference number
Traditional cardiovascular risk factors		
Hypertension	Observational–prospective Observational–cross-sectional Observational–retrospective	20 19 18, 22, 23
Diabetes mellitus	Registry data Observational–retrospective Consensus meeting	27 28, 29, 30 32
Dyslipidaemia	Observational–retrospective Double-blind RCT Post-hoc analysis of RCT	15, 28 12, 39 37
Smoking	Observational–retrospective Post-hoc analysis of RCT	40 41
Obesity	Systematic review Observational–prospective Observational–retrospective Registry study	44 46 45, 47, 48 43
Non-traditional cardiovascular risk factors		
Renal impairment	Nested case-control within RCT Post-hoc analysis of RCT	49 50
Proteinuria	Systematic review Meta-analysis Observational–retrospective	54 55 53, 56
Left ventricular hypertrophy	Double-blind RCT Observational–prospective Observational–retrospective	62 61 57
Anaemia	Systematic review Observational–retrospective	54 57
Acute rejection	Observational–retrospective	15, 64
Immunosuppression-related cardiovascular risk		
Belatacept use	Systematic review Observational–retrospective	66 65
Cardiovascular risk prediction		
Renal transplant recipients	Systematic review Observational–prospective Observational–retrospective Validation on RCT data	71 69 64, 67, 68, 70 72, 73
Biomarkers of cardiovascular disease	Post-hoc analysis of RCT Meta-analysis Observational–prospective	80 75, 76, 77 78, 79

### Renal impairment

Successful kidney transplantation and the restoration of renal function significantly reduces, but does not negate, the risk of cardiovascular mortality in recipients. Even an allograft with excellent function does not restore to the recipient an entirely normal glomerular filtration rate (GFR). In this sense, transplant recipients should be considered a unique cohort of patients with enduring, albeit less advanced, CKD.

A study by Foster and colleagues demonstrated that estimated GFR (eGFR), calculated from creatinine, cystatin C or β2-microglobulin, is an independent risk factor for cardiovascular events and mortality in RTR [49]. The highest level of incident cardiovascular disease and all-cause mortality is associated with the lowest eGFR. A post hoc analysis of the FAVORIT study suggested that this only becomes relevant once the eGFR falls below 45 ml/min/1.73 m^2^, with no association with incident cardiovascular disease or all-cause mortality above this threshold. However, below this cut-off, each 5 ml/min/1.73 m^2^ increase in eGFR is associated with a 15% reduction in cardiovascular disease and mortality [50].

Such studies have highlighted the importance of achieving, and subsequently maintaining, optimal graft function to favourably modify cardiovascular risk in RTR. Strategies to improve graft longevity have long been considered, (increased use of living donor organs is one option), but there is yet to be consensus on the ideal immunosuppression regimen, which balances the risks of chronic immunologically-mediated damage and calcineurin inhibitor nephrotoxicity [51].

### Proteinuria

Proteinuria is a commonly identified issue in RTR. Its exact prevalence is difficult to ascertain due to the varying thresholds used in studies, but approximately 20% of RTR have proteinuria of greater than 1 g/day [52]. Analogous to its impact in the general population, proteinuria in RTR is associated with cardiovascular disease. A study by Fernandez-Fresnedo and colleagues demonstrated that persistent proteinuria doubles the risk of cardiovascular disease and all-cause mortality in RTR [53].

Despite the widespread use of renin-angiotensin system (RAS) blockade in patients with proteinuric CKD, the evidence-base in the transplant population is not definitive. A systematic review by Hiremath and colleagues, which included 21 trials and 1549 patients, demonstrated that RAS blockade effectively reduces proteinuria in RTR [54]. However, the median follow-up time of 27 months was insufficient to determine effects on graft and patient outcomes. A more recent systematic review by the same group failed to show a survival benefit from use of RAS blockade in RTR [55]. Additionally, a further retrospective study involving over 39,000 recipients demonstrated that use of RAS blockade did not reduce the risk of cardiovascular death compared to other forms of antihypertensive medication [56].

Nevertheless, UK and US guidelines continue to advocate the use RAS blockage in RTR with proteinuria based on their ability to reduce urinary protein excretion [24, 25].

### Left ventricular hypertrophy

Although it may be classified as a form of cardiovascular disease in itself, LVH is an independent risk factor for congestive cardiac failure and mortality in RTR [57]. It is common in RTR and is closely linked to hypertension and anaemia [57].

Several methods exist for the diagnosis of LVH, including electrocardiography (ECG), echocardiography (ECHO), and cardiac magnetic resonance imaging (CMRI) [58]. At present, use of CMRI for this purpose is not routine due to cost considerations and labour intensity. ECHO is subject to operator variability but is considered the gold-standard modality. It is portable, widely available and has been repeatedly used in both observational and interventional studies in RTR [58, 59]. When ECHO is used, LV mass must be corrected for body size either by indexing to body surface area (BSA) or height raised to the allometric power of 2.7 (height^2.7^). Both indexes have been used in studies involving RTR, and joint recommendations by the American Society of Echocardiography and the European Society of Cardiovascular Imaging do not favour use of one over the other. However, height^2.7^ may have advantages in obese patients, which makes this index more applicable to RTR where obesity is common [60]. Although it is possible to diagnose LVH by ECG (and this diagnosis is associated with mortality in RTR), lack of sensitivity means ECG should be considered second-line [57, 58].

In one study, regression of LVH in RTR reduced the risk of cardiovascular events by 59% [61]. A randomised trial by Midtvedt and co-workers demonstrated that a functioning allograft and well-controlled blood pressure lead to LVH regression [62]. In this study, ACE inhibitors and calcium-channel blockers were equally effective. Thus, achieving adequate blood pressure control should be viewed as more important than the agent used.

Increasingly, mammalian target of rapamycin (mTOR) inhibitors have been investigated as potential promoters of LVH regression. The mTOR signalling pathway is involved in modulating the cardiac response to haemodynamic stress. Several small randomised controlled trials in RTRs have shown reduction in LV mass with these agents used as immunosuppression [59]. Much larger randomised controlled trials will be required to confirm these findings and investigate the impact on survival before mTOR inhibitors are routinely used for their cardiac remodelling properties.

## Other non-traditional risk factors

Several other risk factors for cardiovascular disease which are common in transplant recipients have been identified. Anaemia affects 20–45% of RTR [63]. It is attributed to a combination of factors including suboptimal graft function, RAS blockade, antiproliferative agents, mTOR inhibitors and co-trimoxazole [54, 63]. Akin to its impact in the CKD population, post-transplant anaemia is associated with the development of LVH and congestive heart failure [57]. Its impact on patient survival remains unclear [63]. Treatment in RTR should be analogous to management of anaemia in CKD patients [25]. However, caution must be exercised as over-correction has also been associated with increased mortality risk in RTR [63].

A study by Kasiske and co-workers demonstrated that two or more episodes of acute rejection in the first year increases the risk of ischaemic heart disease by 62% [15]. The PORT study also found acute rejection associates with an increase in cardiovascular risk [64]. The underlying mechanism for this association remains unclear. It is possible that the immune activation which occurs in rejection is involved in the pathogenesis of cardiovascular disease. However, the effects of the additional immunosuppression used to treat rejection may also contribute to increased cardiovascular risk.

## Immunosuppression and cardiovascular risk

Immunosuppression is essential post-transplantation to prevent graft loss from acute rejection and chronic immunological injury. Despite the positive impact immunosuppressant agents have on long-term graft outcomes, their negative effects on RTR are equally important. Steroids and calcineurin inhibitors contribute significantly to the increased risk of cardiovascular disease faced by RTR. This occurs primarily through their amplification of traditional risk factors such as hypertension, diabetes and dyslipidaemia [16, 31, 35]. While these risk factors can be addressed, this is often at the expense of using additional medications such as antihypertensives, insulin and statins. Therefore, steroid or calcineurin inhibitor avoidance has been suggested as an alternative strategy to reduce cardiovascular risk. Trial evidence to define the optimal immunosuppression regimen for this purpose is lacking and the potential for increased risk of rejection must be borne in mind. Guidelines by the Renal Association in the UK advocate that a flexible approach to immunosuppression is required, with RTR being stratified by risk [25].

In recent years, attempts have been made to develop novel immunosuppressive agents to allow calcineurin inhibitor avoidance without risk of allograft rejection. Treatment with one such drug, belatacept, has been associated with an improvement in graft function, blood pressure and lipid profile, and a lower incidence of post-transplantation diabetes [65, 66]. However, these effects have not yet been demonstrated to lead to an improvement in the long-term cardiovascular risk profile of belatacept treated-recipients.

## Predicting cardiovascular risk

Various strategies to address the worldwide prevalence of cardiovascular disease have appropriately focused on prevention rather than cure. Such an approach requires methods to identify at-risk individuals for targeted cardiovascular risk reduction. In the general population, various prediction models for cardiovascular risk have been developed for this purpose.

One potential pitfall when using cardiovascular risk scores is that they may under- or overestimate risk when used in an inappropriate patient group. Risk scores are validated for use only on the population from which they were derived. For example, the Framingham cohort were a homogenous group of individuals, predominantly white and middle-aged. Therefore, despite various updates and adjustments, the Framingham risk score is limited in its ability to predict cardiovascular risk in the various ethnic groups worldwide. This was the primary motivation for the development of QRISK and subsequently QRISK2 scores [67].

Several studies on cardiovascular risk prediction in RTR have applied the Framingham risk score. These studies demonstrated that the risk score appropriately identifies recipients at low-risk. However, it greatly underestimates cardiovascular risk in recipients at moderate and high-risk [68–71]. Therefore, recipients in most need of effective preventative strategies may be overlooked.

This underestimation of risk in transplant recipients is partly because non-traditional risk factors play an important role but are unaccounted for in general population-based scores [69].

### Transplant-specific cardiovascular risk prediction

Several large studies have attempted to develop risk prediction models specifically for use in RTR. In the PORT study, Israni and co-workers analysed both traditional and non-traditional risk factors for cardiovascular disease in over 23,000 RTR [64]. They showed that important risk factors for prediction included age, sex, race, pre-existing diabetes, post-transplant diabetes, pre-existing cardiovascular disease, eGFR, acute rejection, delayed graft function and duration of ESRD. Further analysis revealed that traditional risk factors added little to the accuracy of the model. The non-traditional factors included in their risk prediction score were closely related to graft function.

In 2012, Soveri and colleagues developed the cardiovascular risk calculator for renal transplant recipients (CRCRTR-MACE), which provides an estimate of risk over 7 years [72]. This calculator was established using seven variables including age, previous ischaemic heart disease, diabetes, LDL-cholesterol, serum creatinine, number of previous transplants and smoking status. The authors proceeded to validate its use in RTR using individuals in an international database and two clinical trials [73]. When compared to Framingham risk score, the CRCRTR-MACE more accurately predicted the risk of cardiovascular events in RTR. The improvement in risk prediction with CRCRTR-MACE appears to be primarily related to the addition of GFR to traditional risk factors [68].

### Biomarkers of cardiovascular disease

Risk prediction models based on traditional cardiovascular risk factors remain the cornerstone of effective prevention. However, these models have limitations and fail to identify every individual who will develop cardiovascular disease. Attempts have therefore been made to improve their accuracy through the addition of other risk markers, often termed “biomarkers.” In the general population, a vast array of biomarkers of cardiovascular disease has been identified, with the utility of up to 30 markers being investigated in one study [74]. A single biomarker is unlikely to outperform and therefore replace an existing risk prediction model. Rather, biomarkers are studied as potential adjuncts to be incorporated into established models.

Several widely-used cardiovascular biomarkers, measured in serum, have been extensively studied in patients with renal disease. The concentrations of Troponin T (TnT) and N-terminal B-type natriuretic peptide (NT-proBNP) are strongly correlated with eGFR and as a result, their interpretation can be challenging. Despite this, both biomarkers appear to retain their predictive value in various groups of patients with renal disease. Elevations of TnT are associated with all-cause and cardiovascular mortality in patients with CKD [75]. In 2013, a meta-analysis including 27 studies found an association between elevated serum NT-proBNP concentration and cardiovascular mortality in ESRD patients [76].

C-reactive protein (CRP) may also be useful as a prognostic biomarker in patients with ESRD. A systematic review of 109 studies found that CRP concentration was strongly associated with cardiovascular death in dialysis patients [77].

Research into the use of circulating cardiovascular biomarkers specifically in RTR has been more limited. A study by Connolly and co-workers demonstrated that TnT concentration is a strong predictor of cardiovascular mortality in this population [78]. Another study showed that the pre-transplant TnT concentration was predictive of survival in the post-transplant period. Higher concentrations were associated with increased risk of fatal cardiovascular events [79]. A large study with over 2000 participants by Abedini and colleagues found that CRP is similarly associated with major cardiovascular events in stable RTR [80]. Despite the confounding influence of renal function on the concentrations of TnT and NT-proBNP, these and other circulating biomarkers may be suitable adjuncts to existing risk prediction models for RTR.

Several recent reviews have also highlighted the potential use of physical biomarkers of cardiovascular disease for improving the accuracy of risk prediction [81, 82]. For example, pulse wave velocity (PWV) is a non-invasive measure of arterial stiffness and has been repeatedly shown to independently predict cardiovascular events and mortality in RTR [81]. Similarly, coronary artery calcification (CAC) scores obtained from cardiac computed tomography (CT) have been demonstrated to act as strong predictors of mortality in this population [82]. Further work is therefore required to clarify which biomarkers are most useful and how they should be incorporated into clinical practice.

## Conclusion

Despite improvements in cardiovascular risk management and outcomes, cardiovascular disease remains a leading threat to graft and patient survival following renal transplantation. In RTR there exists a unique combination of traditional and non-traditional cardiovascular risk factors. The accelerated risk of cardiovascular disease is often amplified by the effects of immunosuppression. As a result, risk prediction models developed in the general population often under-estimate risk in RTR. The use of transplant-specific risk calculators may go some way towards overcoming this problem. Addition of cardiovascular biomarkers to these scores may improve accuracy but further work in this area is required. At present, aggressive management of traditional risk factors remains the cornerstone of effective cardiovascular disease prevention. However, other strategies to prolong optimal graft function and overcome the negative aspects of current immunosuppression regimens should also be considered.

## Electronic supplementary material

Below is the link to the electronic supplementary material.

Supplementary material 1 (DOCX 12 KB)

## Data Availability

Data sharing is not applicable to this article as no datasets were generated or analysed during the current study.
