# Supplementary material for: Cardiovascular risk in renal transplant recipients
Source: J Nephrol. 2018 Nov 7;32(3):389–99. doi: 10.1007/s40620-018-0549-4 (PMC6482292; doi:10.1007/s40620-018-0549-4)
Supplement: Supplementary file 1 — Supplementary material 1 (DOCX 12 KB) [file 40620_2018_549_MOESM1_ESM.docx]

**Appendix 1: Search strategy**

To identify the studies and literature relevant to this comprehensive, narrative review, we performed an electronic search of MEDLINE from the date of database inception until September 2017. There was no language restriction. Search terms and key words used included: “kidney transplantation”, “dialysis”, “chronic kidney disease”, “end-stage renal disease”, “mortality”, “cardiovascular mortality”, “cardiovascular disease”, “myocardial infarction”, “myocardial ischemia”, “stroke”, “peripheral vascular disease”, “hypertension”, “diabetes mellitus “, “dyslipidaemia”, “smoking”, “obesity”, “proteinuria”, “left ventricular hypertrophy”, “troponin”, “c-reactive protein”, “natriuretic peptides”, “biomarkers”.

We manually reviewed the reference list of all retrieved studies to identify other relevant studies which had not been included. Abstracts were reviewed for relevance prior to exclusion of studies. Guidelines relevant to the long-term care of renal transplant recipients were also accessed from KDIGO and Renal Association websites.
